# Supplementary material for: Structure-guided peptide engineering of a positive allosteric modulator targeting the outer pore of TRPV1 for long-lasting analgesia
Source: Nat Commun. 2023 Jan 3;14:4. doi: 10.1038/s41467-022-34817-1 (PMC9810691; doi:10.1038/s41467-022-34817-1)
Supplement: Supplementary file 1 — Supplementary Information [file 41467_2022_34817_MOESM1_ESM.pdf]

Supplementary information for

Rational design of a positive allosteric modulator targeting the outer pore of TRPV1 for long-lasting analgesia

Heng Zhang [1, 2, 6, †], Jia-Jia Lin [2, 3, †], Ya-Kai Xie [2, 3], Xiu-Zu Song [4], Jia-Yi Sun [4], Bei-Lei Zhang [4], Yun-Kun Qi [5, *], Zhen-Zhong Xu [2, 3, *], Fan Yang [1, 2, 6, *]

[1] Department of Biophysics, Kidney Disease Center of the First Affiliated Hospital, Zhejiang University School of Medicine, Hangzhou, Zhejiang Province, 310058, China.

[2] NHC and CAMS Key Laboratory of Medical Neurobiology, MOE Frontier Science Center for Brain Research and Brain-Machine Integration, School of Brain Science and Brain Medicine, Zhejiang University, Hangzhou, Zhejiang, 310058, China.

[3] Department of Neurobiology and Department of Anesthesiology of First Affiliated Hospital, Zhejiang University School of Medicine, Hangzhou, Zhejiang, 310058, China

[4] Department of Dermatology, The Third People's Hospital of Hangzhou, Zhejiang, Hangzhou, China.

[5] Department of Medicinal Chemistry, School of Pharmacy, Qingdao University, Qingdao 266021, Shandong, China.

[6] Alibaba-Zhejiang University Joint Research Center of Future Digital Healthcare

[*] Corresponding author. Email: Email: qiyunkun@qdu.edu.cn;; xuzz@zju.edu.cn; fanyanga@zju.edu.cn

[†] These authors contributed equally to this work.

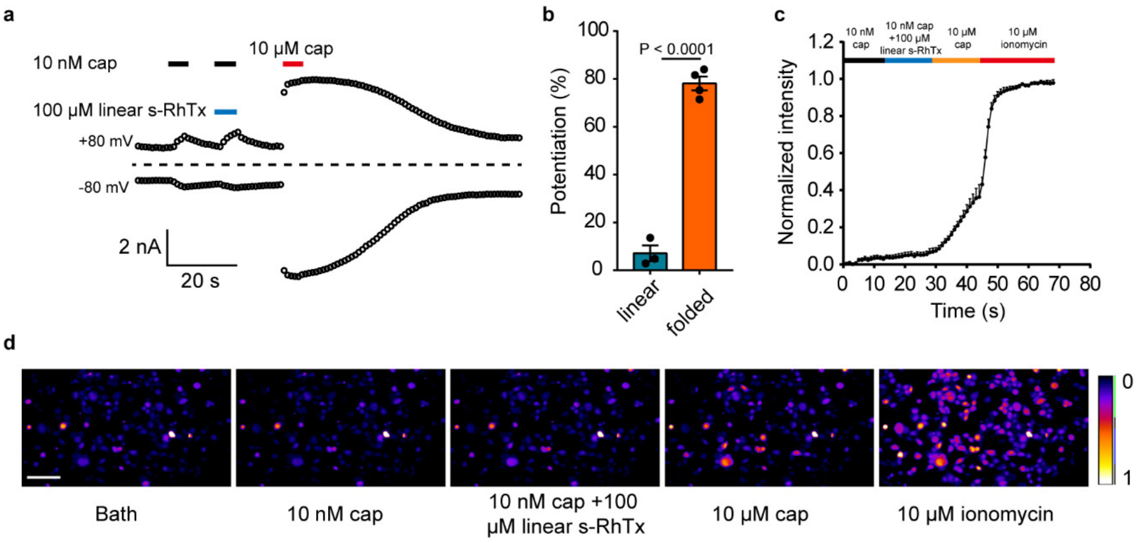

Supplementary Fig. 1: Linear s-RhTx barely potentiates in the activation of 10 nM capsaicin on TRPV1.

a whole-cell current of TRPV1 responded to 10 nM capsaicin, the mixture of 10 nM capsaicin and 100 μM linear s-RhTx and 10 μM capsaicin. **b** Comparison of the potentiation ratio for 100 μM linear or 100 μM folded s-RhTx on the activation of TRPV1 by 10 nM capsaicin (data was shown as mean ± S.E.M., n=3 for linear and for folded, n = 4 biologically independent cells; two-sided t-test, P < 0.0001, t = 16.2, df = 5). **c** the normalized fluorescence intensity of calcium imaging (means ± S.E.M.; n = 6 biologically independent cells). **d** Representative calcium imaging in HEK293T cells expressing TRPV1 responded to 10 nM capsaicin, the mixture of 10 nM capsaicin and 100 μM linear s-RhTx, 10 μM capsaicin and 10 μM ionomycin, respectively, scale bar = 100 μm.

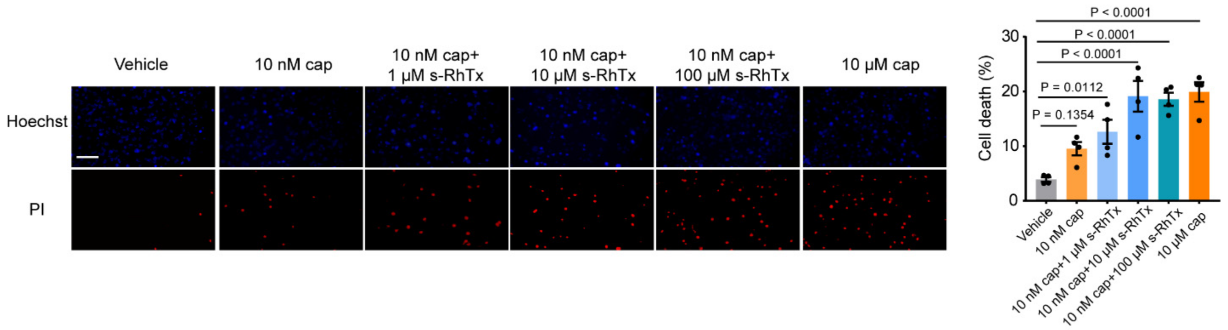

Supplementary Fig. 2: Cell death assay by Hoechst and PI.

Left panel, images of HEK293T cells stained with Hoechst (top panel) for nuclei in all cells and PI (bottom panel) for nuclei in dead cells. Scale bar = 100 µm. Right panel, bar graph of cell death ratio (means ± S.E.M.; n = 4 biologically independent cells). One-way ANOVA, F (5, 18) = 13.11; P (Veh vs 10 nM cap) = 0.1354, P (Veh vs 10 nM cap + 1 µM s-RhTx) = 0.0112, P (Veh vs 10 nM cap + 10 µM s-RhTx) <0.0001, P (Veh vs 10 nM cap + 100 µM s-RhTx) < 0.0001, P (Veh vs 10 nM cap + 10 µM cap) < 0.0001.

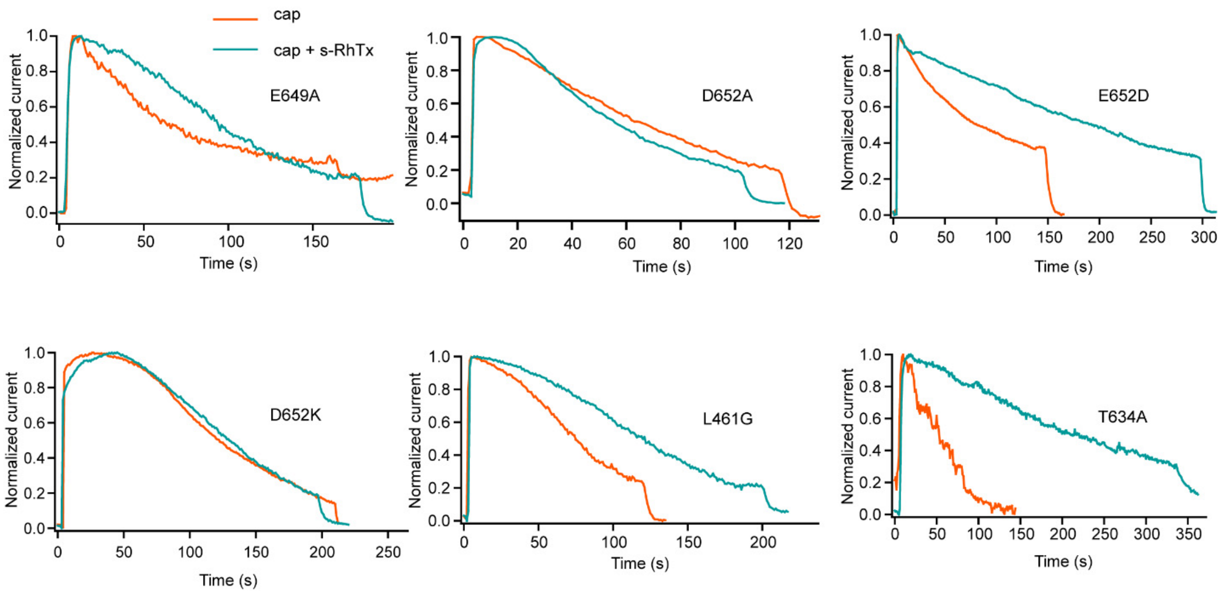

Supplementary Fig. 3: Representative current desensitization traces of TRPV1 mutants responded to capsaicin or the cocktail of capsaicin and s-RhTx in the presence of calcium at +80 mV.

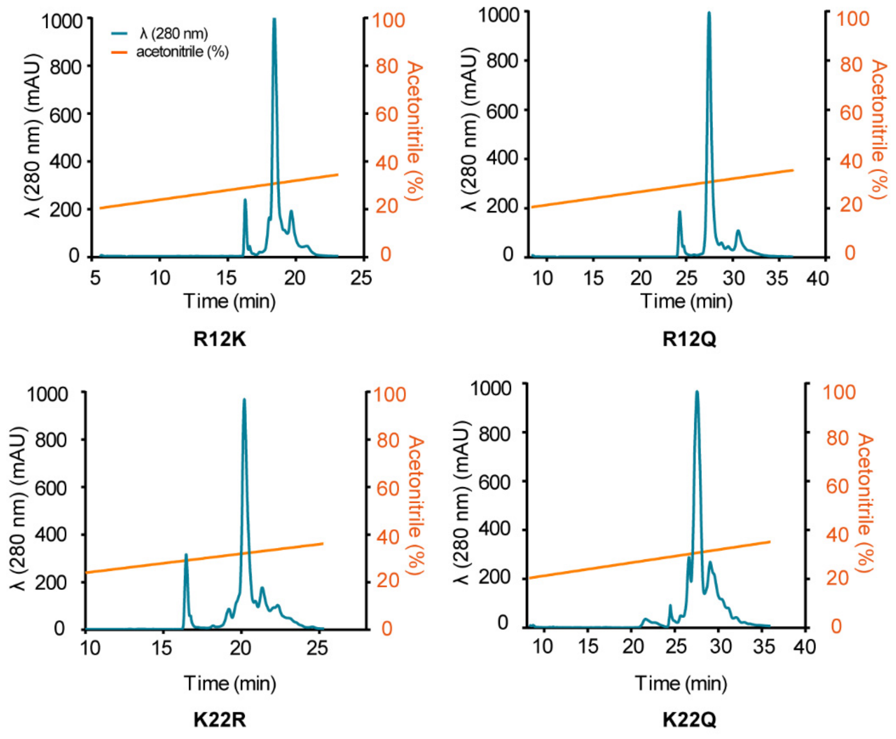

Supplementary Fig. 4: Purification of s-RhTx mutants by reverse phase chromatography.

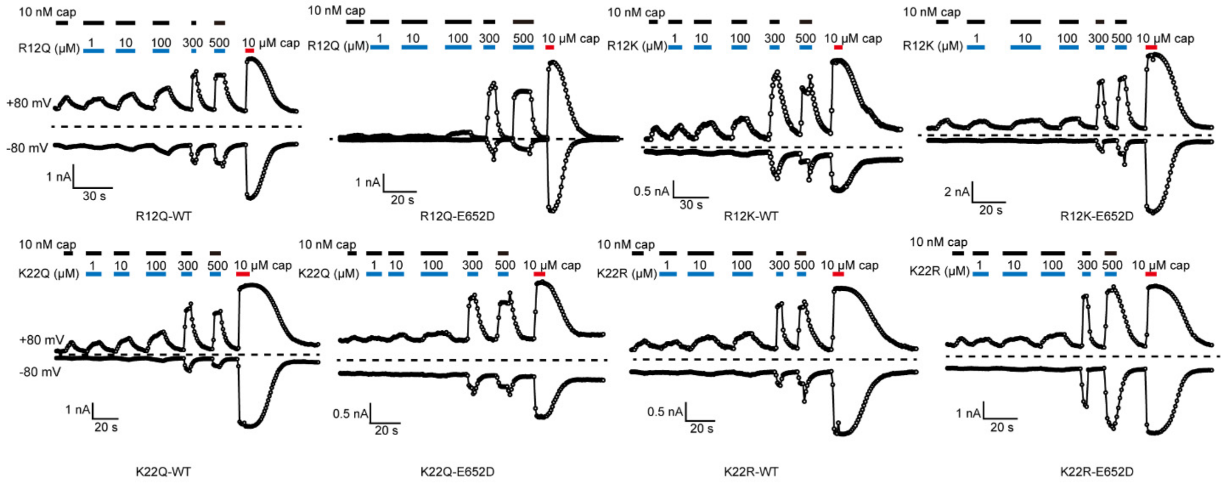

Supplementary Fig. 5: Concentration-response curves of s-RhTx mutants on WT TRPV1 and TRPV1 E652D mutant.

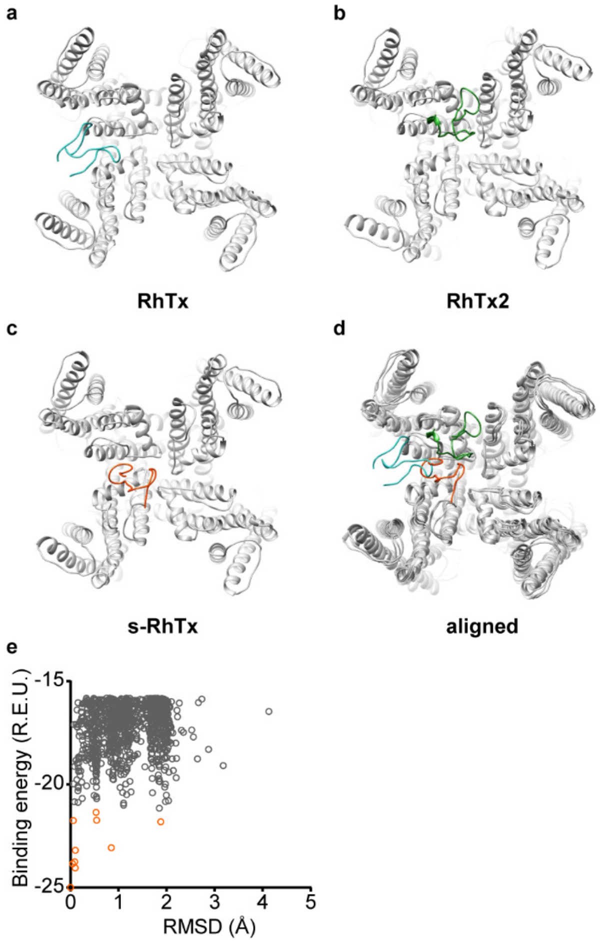

Supplementary Fig. 6: Docking models of RhTx, RhTx2 and s-RhTx to TRPV1 channel.

a, **b** and **c**, top view of docking model of RhTx (in cyan), RhTx2 (in green) and s-RhTx (in orange) to TRPV1 channel. **d** Alignment of docking results of RhTx, RhTx2 and s-RhTx to TRPV1 channel. **e** Plot of the root mean square deviation (RMSD) of top 1000 models with lowest-binding energy versus their binding energies in Rosetta energy unit (REU). Top 10 models with lowest-binding energy used for further analysis were labeled in orange.

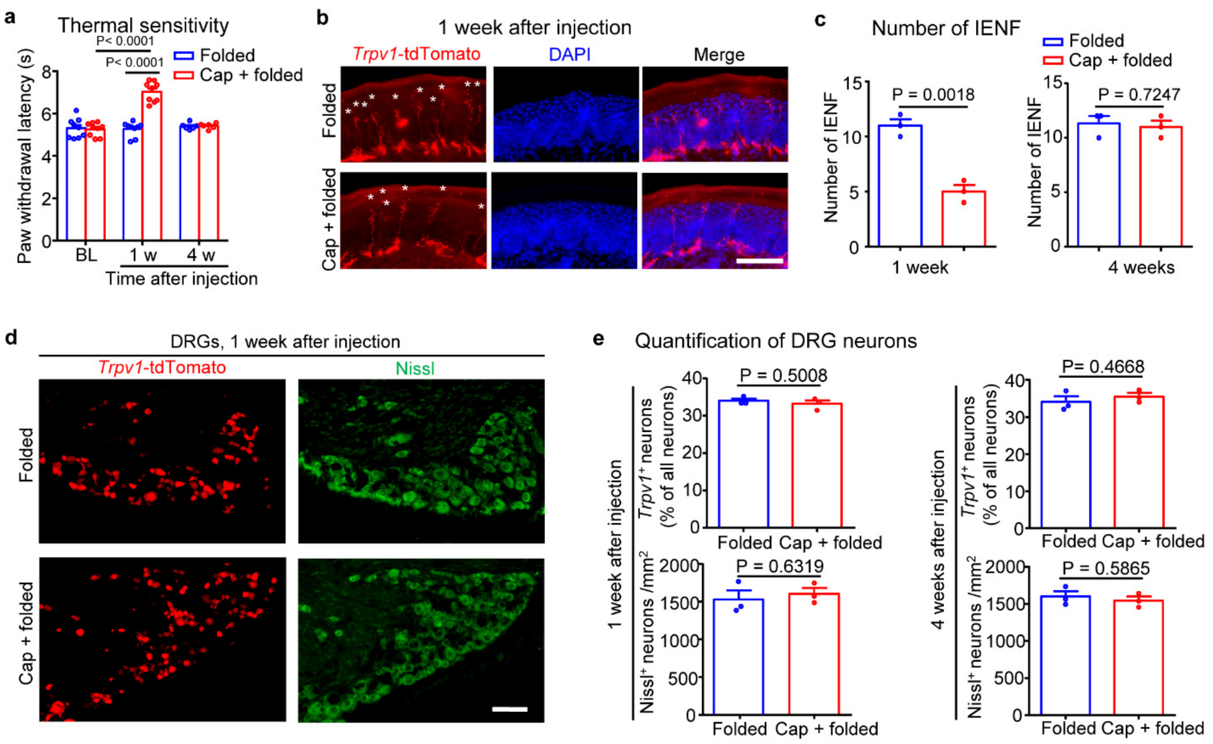

Supplementary Fig. 7: Co-application of s-RhTx with low-dose capsaicin induced the reversible IENF degeneration but not the loss of DRG neurons.

a Thermal sensitivity of mice with the intraplantar injection of folded s-RhTx (2 μg) with or without capsaicin (200 ng). BL and 1 week (w) n = 9, 4 w n = 6 mice biologically independent per group. P value were labeled in the figure, two-way ANOVA followed by Bonferroni's post hoc test. **b** Representative images showing *Trpv1*$^+$ nerve fibers degeneration in epidermis from *Trpv1-Ai14* mice after the treatment by folded s-RhTx with capsaicin. Scale bars, 100 μm. Every asterisk represents one fiber. Tissue sections were collected from three mice, and three independent immunofluorescence experiments were conducted with similar results. **c** Quantification of *Trpv1*$^+$ IENF in epidermis at 1 week (left) or 4 weeks (right) after the treatment with s-RhTx and capsaicin. Two-sided unpaired student's t test, n = 3 biologically independent mice per group. At 1 week, t = 7.348, df = 4; at 4 w, t = 0.3780, df = 4. **d** Representative immunohistochemical images showing *Trpv1*$^+$ and Nissl$^+$ neurons in DRGs from *Trpv1-Ai14* mice after the treatment by folded s-RhTx with or without capsaicin. Three

independent immunofluorescence experiments were conducted with similar results. **e** Quantification of *Trpv1*$^+$ and Nissl$^+$ neurons in DRGs at 1 week (left) or 4 weeks (right) after the treatment by folded s-RhTx and capsaicin. Two-sided unpaired student's t test, n = 3 biologically independent mice per group. *Trpv1*$^+$ neurons at time point of 1 w, t = 0.7392, df = 4; *Trpv1*$^+$ neurons at 4 w, t = 0.8034, df = 4; *Nissl*$^+$ neurons at 1 w, t = 0.5178, df = 4; *Nissl*$^+$ neurons at 4 w, t = 0.5906, df = 4. Scale bars, 100 μm. All data were shown as mean ± S.E.M..

Name	Primer forward	Primer reverse
L461G	gaaggcggacccccctataagctgaataacaccgt	ggggccagccttccacaggccgatagtaa
D602A	ctgatcgaggctgggaagaataactcactgcc	tcccagcctcgatcagtgtcactacg
K604A	gatgggacgaataactcactgcctgtggag	gagttattcgtcccatcctcgatcagtgt
K604E	gatggggagaataactcactgcctgtggag	gagttattctccccatcctcgatcagtgtcac
T634A	ctgtattccgcatgtctggagctgttcaag	ctccagacatgcggaatacaggctgttgtaaga
R637A	catgtctggcgctgttcaagttcaccatcg	cttgaacagcgccagacatgtggaatacag
R637N	cacatgtctgaacctgttcaagttcaccatcggcatg	cttgaacaggttcagacatgtggaatacaggc
K640T	gctgttcacgttcaccatcggcatgg	gatggtgaacgtgaacagctccagacatg
K640R	gctgttcaggttcaccatcggcatgg	gatggtgaacctgaacagctccagacatg
E649A	gtgacctggcgttcaccgagaactatgact	tgaacgccaggtcacccatgccgat
E652A	ggagttcaccgcgaactatgacttcaaggc	gtcatagttcgcggtgaactccaggtcac
E652K	ggagttcaccaagaactatgacttcaaggctg	gtcatagttcttggtgaactccaggtcacc
E652D	ggagttcaccgacaactatgacttcaaggctg	gtcatagttgtcggtgaactccaggtcacc
K657A	atgacttcacggctgtcttcatcatcctgttac	gaagacagccgtgaagtcatagttctcggtgaac
K657E	atgacttcgaggctgtcttcatcatcctgttac	gaagacagcctcgaagtcatagttctcggtgaac

Table S1 Primers used to generate TRPV1 mutants
