# Supplementary material for: Structure-guided peptide engineering of a positive allosteric modulator targeting the outer pore of TRPV1 for long-lasting analgesia
Source: Nat Commun. 2023 Jan 3;14:4. doi: 10.1038/s41467-022-34817-1 (PMC9810691; doi:10.1038/s41467-022-34817-1)
Supplement: Supplementary file 3 — Reporting Summary [file 41467_2022_34817_MOESM3_ESM.pdf]

nature portfolio

Corresponding author(s): Fan Yang

Last updated by author(s): Oct 31, 2022

Reporting Summary

Nature Portfolio wishes to improve the reproducibility of the work that we publish. This form provides structure for consistency and transparency in reporting. For further information on Nature Portfolio policies, see our Editorial Policies and the Editorial Policy Checklist.

Statistics

For all statistical analyses, confirm that the following items are present in the figure legend, table legend, main text, or Methods section.

n/a	Confirmed	
☐	☒	The exact sample size (*n*) for each experimental group/condition, given as a discrete number and unit of measurement
☐	☒	A statement on whether measurements were taken from distinct samples or whether the same sample was measured repeatedly
☐	☒	The statistical test(s) used AND whether they are one- or two-sided<br>*Only common tests should be described solely by name; describe more complex techniques in the Methods section.*
☒	☐	A description of all covariates tested
☒	☐	A description of any assumptions or corrections, such as tests of normality and adjustment for multiple comparisons
☐	☒	A full description of the statistical parameters including central tendency (e.g. means) or other basic estimates (e.g. regression coefficient) AND variation (e.g. standard deviation) or associated estimates of uncertainty (e.g. confidence intervals)
☐	☒	For null hypothesis testing, the test statistic (e.g. *F*, *t*, *r*) with confidence intervals, effect sizes, degrees of freedom and *P* value noted<br>*Give P values as exact values whenever suitable.*
☒	☐	For Bayesian analysis, information on the choice of priors and Markov chain Monte Carlo settings
☒	☐	For hierarchical and complex designs, identification of the appropriate level for tests and full reporting of outcomes
☒	☐	Estimates of effect sizes (e.g. Cohen's *d*, Pearson's *r*), indicating how they were calculated

Our web collection on statistics for biologists contains articles on many of the points above.

Software and code

Policy information about availability of computer code

Data collection	PatchMaster v2x53;Rosetta version 2019; Ocular Software version 2.0.1.496; Hargreaves radiant heat apparatus;Nikon Eclipse Ti2 microscope with charge-coupled device camera;
Data analysis	Igor Pro 5.0; Office Excel version 2019; UCSF Chimera version 1.1219; Graphpad Prism version 7.0; Fiji image j version 1.53.

For manuscripts utilizing custom algorithms or software that are central to the research but not yet described in published literature, software must be made available to editors and reviewers. We strongly encourage code deposition in a community repository (e.g. GitHub). See the Nature Portfolio guidelines for submitting code & software for further information.

Data

Policy information about availability of data

All manuscripts must include a data availability statement. This statement should provide the following information, where applicable:

- Accession codes, unique identifiers, or web links for publicly available datasets
- A description of any restrictions on data availability
- For clinical datasets or third party data, please ensure that the statement adheres to our policy

All data needed to evaluate the conclusions in the paper are present in the paper. The data that support this study are available from the corresponding authors upon reasonable request. The PDB files used in this study are available in RCSB PDB website (2MVA, https://www.rcsb.org/structure/2MVA; 3J5P, https://www.rcsb.org/structure/3J5P). The source data are provided as the Source Data Files.

Human research participants

Policy information about studies involving human research participants and Sex and Gender in Research.

Reporting on sex and gender	N/A
Population characteristics	N/A
Recruitment	N/A
Ethics oversight	N/A

Note that full information on the approval of the study protocol must also be provided in the manuscript.

Field-specific reporting

Please select the one below that is the best fit for your research. If you are not sure, read the appropriate sections before making your selection.

☒ Life sciences ☐ Behavioural & social sciences ☐ Ecological, evolutionary & environmental sciences

For a reference copy of the document with all sections, see nature.com/documents/nr-reporting-summary-flat.pdf

Life sciences study design

All studies must disclose on these points even when the disclosure is negative.

Sample size	Experiments were performed at least three independent times. Sample size in all experiments were estimated based in previous published experiments (ref. 19,31,34) and power analysis was used to justify the sample size. In this study, the statistic analysis was obtained using student t test and the values represents means plus minus standard error of the mean.
Data exclusions	No data were excluded.
Replication	Since experiments were performed at least three independent times, all experimental findings were reproducible.
Randomization	Samples and organisms were randomly allocated into experimental groups.
Blinding	For experiments in the cell level, results of experiments were so obvious, the blinding was unnecessary. For behavioral test, mice were tested by an investigator blinded to mouse genotype or drug treatment.

Reporting for specific materials, systems and methods

We require information from authors about some types of materials, experimental systems and methods used in many studies. Here, indicate whether each material, system or method listed is relevant to your study. If you are not sure if a list item applies to your research, read the appropriate section before selecting a response.

Materials & experimental systems

n/a	Involved in the study	
☒	☐	Antibodies
☐	☒	Eukaryotic cell lines
☒	☐	Palaeontology and archaeology
☐	☒	Animals and other organisms
☒	☐	Clinical data
☒	☐	Dual use research of concern

Methods

n/a	Involved in the study	
☒	☐	ChIP-seq
☒	☐	Flow cytometry
☒	☐	MRI-based neuroimaging

Eukaryotic cell lines

Policy information about cell lines and Sex and Gender in Research

Cell line source(s)	HEK293T (ATCC, #CRL-3022)
Authentication	No further authentications were performed for this study.

Mycoplasma contamination	No mycoplasma contamination tests were performed for this study.
Commonly misidentified lines (See ICLAC register)	None of the cell lines used are listed in the ICLAC database.

Animals and other research organisms

Policy information about studies involving animals; ARRIVE guidelines recommended for reporting animal research, and Sex and Gender in Research

Laboratory animals	Adult C57 BL/6 mice (male, 8–10 weeks) and Trpv1-/- mice (Jax Strain #:003770) were used for behavioral studies.To specifically label Trpv1-positive nerve fibers in skin tissues, Trpv1-Cre mice (Jax Strain #:017769) were crossed with floxed reporter lines (Ai32, Jax Strain #:012569 or Ai14, Jax Strain #:007914) to get the transgenic reporter (Trpv1-Ai32 or Trpv1-Ai14) mice. Mice were group-housed at 22–25 °C and relative humidity of 30–70% under the 12 h light-dark cycle with access to standard food and water ad libitum.
Wild animals	No wild animals were used in this study.
Reporting on sex	In order to avoid the influence of physiological cycle and hormone level changes on pain perception behavior, female mice were not used in behavioral experiments. Other experiments included male and female animals.
Field-collected samples	No field-collected samples were used in this study.
Ethics oversight	All animal studies and experimental procedures were approved by the Animal Care and Use Committee of the animal facility at Zhejiang University.

Note that full information on the approval of the study protocol must also be provided in the manuscript.
